# 2020 Editor’s Choice Articles in the “Cell Nuclei: Function, Transport and Receptors” Section

**DOI:** 10.3390/cells11172625

**Published:** 2022-08-24

**Authors:** Hiroshi Miyamoto

**Affiliations:** 1 Department of Pathology & Laboratory Medicine, University of Rochester Medical Center, Rochester, NY 14642, USA; hiroshi_miyamoto@urmc.rochester.edu; 2 Department of Urology, University of Rochester Medical Center, Rochester, NY 14642, USA; 3 James P. Wilmot Cancer Institute, University of Rochester Medical Center, Rochester, NY 14642, USA

In 2020, a total of 106 original research articles, 84 reviews, and 1 other paper were published within the “Cell Nuclei: Function, Transport and Receptors” section. Of these, I selected nine papers as Editor’s Choice Articles. Herein, I summarize the observations demonstrated in the selected studies.

Tau protein has been implicated in the development of neurodegenerative disorders [1], including Alzheimer’s disease, which are often associated with symptoms of depression. However, it remains poorly understood if tau protein is directly involved in depressive symptoms. Criado-Marrero et al. [2] investigated the role of tau in the emergence of depression-like symptoms, using *Mapt*^−/−^ mice carrying a deletion of the gene encoding tau. Compared with wild-type littermates, the knockout animals showed reduced immobility in the tail suppression test and forced swim test, as well as reduced escape failure and latency in the learned helplessness task, indicating more resistance to depressive-like behavior. In addition, tau deletion was found to result in inducing neurogenesis in the hippocampal dentate gyrus and subventricular zone, as well as in the frontal cortex and amygdala. These findings suggest that tau could regulate the symptoms associated with neurodegenerative diseases.

Chemoresistance has been a major challenge in the treatment of advanced breast cancer. Meanwhile, high chromosomal instability, one of the hallmarks of triple-negative breast cancer, has been associated with tumor progression and therapeutic resistance [3]. Indeed, homologous recombination, which prevents genomic instability, has been studied in breast cancer. Meyer et al. [4] investigated if genetic alterations could lead to a repair defect in relation to chemoresistance in breast cancer. Sensitivity to mitomycin C, a chemotherapeutic agent, was shown to correlate with DNA damage foci formation in the S phase. Moreover, the activation of CHEK1 induced resistance to mitomycin C. CHEK1-mediated damage response could prevent replication stress, and ATR-CHEK1 signaling could thus compensate for the reduced function of homologous recombination and errors in double-strand break repair.

Alterations in the *LMNA* gene encoding the nuclear envelope protein lamin A/C have been linked to the development of various muscular disorders, including Emery–Dreifuss muscular dystrophy (EDMD) and LMNA-related congenital muscular dystrophy (L-CMD) [5]. However, the pathophysiology of these striated muscle laminopathies, particularly its differences among specific types, remains to be further explored. Bertrand et al. [6] performed in silico analyses of the UMD-LMNA mutation database and found that mutations in the residues involving the interactions of lamin dimer and tetramer were seen significantly more often in L-CMD patients. Correspondingly, lamin A/C expression was considerably up-regulated in fibroblasts from L-CMD patients, compared with those from control or EDMD patients. In addition, the cell proliferation of myoblasts derived from an L-CMD mouse model was increased, compared with that from wild-type littermates or EDMD mice. L-CMD myoblasts also demonstrated the defects in their differentiation, and the expression of nuclear envelope transmembrane proteins required for chromatin remodeling was down-regulated. Inappropriate lamin A/C assembly is thus suggested to play a key role in the pathogenesis of L-CMD. 

Biotinylation techniques, including biotin protein labeling-based BioID, have been widely utilized for determining the proximity of molecules and identifying protein–protein interaction partners [7]. However, there are some limitations in the application of BioID (e.g., long labeling period, inefficiency at <37 °C). Recently, new ligases, including TurboID and miniTurbo, have been developed, allowing only 10-minute labeling with biotin. To further explore potential practical applications of TurboID and its merits and demerits, May et al. [8] developed stable cell lines expressing BioID and TurboID fusion proteins. Compared with BioID, TurboID enabled robust biotinylation, despite being associated with protein instability, leading to possible self biotinylation, as well as cell toxicity. Further modifications to the currently available ligases or novel biotinylation approaches may thus be required.

Endothelial cell damage is considered to be a key event in the development of atherosclerosis. Meanwhile, it has been documented that progerin, a truncated protein pathognomonic for Hutchinson–Gilford syndrome, could modulate endothelial function [9]. Bidault et al. [10] further assessed the functional role of progerin in primary cultures of human coronary endothelial cells. The overexpression of progerin resulted in an induction of endothelial cell dysfunction characterized by increased inflammation, oxidative stress along with persistent DNA damage, an elevated expression of cell cycle arrest proteins, and cellular senescence. In line with these data, the inhibition of progerin prenylation at least partially restored endothelial function. These findings suggest the direct involvement of progerin in atherogenesis.

Amyloid beta (Aβ) peptides are well known to contribute to the pathogenesis of Alzheimer’s disease. Biological functions of circular RNAs (circRNAs) abundantly expressed in the brain have been studied in some neurodegenerative disorders, including Alzheimer’s disease [11]. Mo et al. [12] identified a circRNA containing the Aβ coding region, circAβ-a, which was expressed in all the examined brains from not only Alzheimer’s patients but also non-dementia controls. circAβ-a could then be translated into a novel Aβ-containing Aβ175 polypeptide in cultured cells and human brain, and Aβ175 was further processed into Aβ peptides. These results indicate the presence of an alternative pathway of Aβ generation.

In addition to original research papers, three review articles were selected as Editor’s Choice Articles. Conti et al. [13] summarized and discussed the role of microRNAs in cell–cell communications with a focus on the cancer microenvironment, as well as in the pathogenesis of multicomplex disorders such as Alzheimer’s disease and obesity. Constâncio et al. [14] summarized and discussed available DNA methylation-based tests in liquid biopsies as biomarkers of the most frequently diagnosed malignancies, including lung cancer, breast cancer, prostate cancer, and colorectal cancer. Jans and Wagstaff [15] summarized and discussed the antiviral activity of an anti-parasitic agent ivermectin, with an emphasis on its efficacy against COVID-19.
